# To Mr. Dunning, Plymouth Dock

**Published:** 1803-10-01

**Authors:** 


To Mr. DUNNING, Plymouth Dock.

My dear Sir,

FROM the consideration of your having been one of those valuable members, who have contributed towards the almost general adoption of the Vaccine pocks, and of the zeal you displayed in overcoming by your writings and practical proofs, the prejudices that existed against so beneficial a discovery, I conceived the following translations from some Spanish manuscripts, respecting the discovery and effects of the goat pocks, would not be unacceptable to you; as it evidently shews how far the zeal of a few can go in remedying the evils of human life, and the benefit that may be expected from such laudable examples. The goat pocks were discovered as far back as February last, but from its not having been proved to equal the vaccine, I forbore to give you any information relative to this subject, until I was properly informed of its good effects. The discoverer of this new species of pocks, is the Hebrew Professor of the Royal College of San Isidro, of this town; and as I am intimately acquainted with him, having known him these eight months, he has been so good as to offer his assistance in furnishing with every information any of my friends, who may wish to consult him on the subject. Should you then have any inquiries to make respecting the goat pocks; besides, if you wish to propose any medical questions concerning any branch of physic as practised in this country, this gentleman has assured me, he will punctually answer them. From the present uncertainty of affairs, and from the far advancement of summer, I shall, in all human probability, leave this place in the course of seven or eight weeks, or perhaps sooner; therefore, you have the choice of either writing to the gentleman himself, or to me, under his address, which I have subjoined, who in case of my having left Madrid, will inform himself of the contents of your letter, and forward to you, as soon as possible, the required information.

I am, &c.

*Madrid, July* 8, 1803. W. A.

---

No. 1.

To his Excellency, Peter Cevallos, &c.

My much esteemed Sir,

AS the health and happiness of the people is the supreme law, I cannot omit troubling your excellency's attention with a discovery which has for its object the benefit of mankind in general, and of this nation in particular. As there are no cows to be found here with pocks for the purpose of inoculation; and on account of this, the faculty find themselves reduced to the necessity of procuring vaccine matter from other kingdoms and provinces, to the detriment of the public health, arising from different causes; so that this valuable discovery could not produce in this kingdom the same happy effects, which have attended it in other countries; furthermore, having discovered in the vicinity of Madrid some goats to have the pocks, the matter of which I found by experience to have the same quality, and to produce the same effects in inoculation and preservation, as the vaccine matter; in consideration that we have fresh matter in this country in abundance, it will most assuredly produce more beneficial effects, than the matter introduced from foreign countries, of whose legitimacy no further security can be obtained, than the assertion of the person who sends it; also extracting matter from one person for the inoculation of another, may also beattended with inconveniences. Your Excellency ought to inform the Royal Medical Society of the purport of this letter, as this body can make the necessary experiments

2 pro pro and contra; and should it turn out, as I flatter myself it will, I shall have the satisfaction of having contributed in some measure to the preservation of some of our species, which is the greatest happiness I can possibly enjoy; and obeying the commands of your Excellency, I remain your most stedfast friend, and assured humble servant,

*Royal College of San Isidro, Madrid, Feb.* 18, 1803. JOHN JOSEPH HEYDECK.

No. 2.

To Mr. John Joseph Heydeck.

Sir,

I received your letter of the 18th inst. which informed me of your having discovered different goats of this country, with pocks the same which cows are known to have; the matter being of the same quality, and producing the same effects by inoculation; which discovery I have laid before the Royal Society of Physicians for their investigation; and have to thank you for the zeal you have shewn for the public welfare. I shall always be your most stedfast friend and assured humble servant,

*Aranjuez,* Feb.* 25, 1803, PETER CEVALLOS.

No. 3.

*His Excellency Mr.* Peter Cevallos, *First Secretary of State, and Privy Counsellor of his Majesty, has communicated to the Royal College of Physicians, the following Royal Order:*

Mr. John Joseph Heydeck, Professor of the Royal College of San Isidro, has communicated to me the circumstance of his having lately observed in different goats in the neighbourhood of Madrid, pocks the same as cows are known to have; and having taken matter from them, found it to possess the same quality, and to produce the same effects in inoculation; so that he believes this discovery may be very beneficial to this country, as there is a scarcity of cows, and perhaps there are not any with the pocks; the faculty being, in consequence, reduced to the necessity of procuring vaccine matter from foreign countries, losing by this means some of its power, to the pre-

* Aranjuez, the King of Spain's country seat, seven leagues distant from Madrid.

judice of those inoculated with it, I communicate the same to you for the information of the Royal College of Physicians, in order that they may make a proper examination of this important discovery. I remain your most stedfast friend, and assured humble servant,

*Aranjuez, Feb.* 23, 1803. PETER CEVALLOS.

To Mr. John Joseph Heydeck.

In obedience to his Majesty's commands, the Royal Medical Society have thought proper to forward the official letters, accompanied with the royal order of his Majesty, to Doctors Ignatius Maria, Luiz de Luzuriaga, and to Mr. Peter Hernandez, physicians to his Majesty, that agreeably to your information, they may be pleased to make the necessary experiments, and to acquaint the Society with the results. The purport of which letter I herewith inform you, and shall always remain your most stedfast friend and assured humble servant,

MANUEL CORGULLO.

*An Account of an Inoculation tried with Matter taken from Goats, on two Children, by the Physicians commissioned by the Royal College of Physicians, from the 26th of May,* 1803.

In consequence of the instructions which I gave to different goat-herds of Madrid, and its neighbourhood, respecting pustules, which, from time to time, are accustomed to appear on the teats of goats, I was advised by Manuel Lopez, goat-herd in the street of S. Bruno, opposite the Royal College of San Isidro, who had some goats with the pock, of the same quality which I described to him on the 26th of May.

May 27. I went to the house of the said goat-herd, and discovered that the goats really had the pustules I was in quest of, one in particular, whose pustules were full of virulent matter, and fit to extract for inoculation; of which I informed the commissioners of the Royal College of Physicians, Mr. Ignatius de Luzuriaga, and Mr. Peter Hernandez. At seven o'clock in the evening they attended, accompanied by Mr. Nicholas Diez, surgeon: and on examining the pustules, found them full of virulent matter, and fit for inoculation. They practised the greatest diligence to find a subject for inoculation, but not finding one, the surgeon, Mr. Nicholas Diez, took matter from the goat, and put it between two pieces of glass for its preservation.

The

The goat, according to my observation, shewed no visible symptoms of pain; her milk was plentiful, and excellent; she eat well, and laboured under no inconveniency from the pustules.

May 28. The wife of the goat-herd acquainted me that she had met with two poor women, who, for a gratification, would permit their children to be inoculated with the goat-pocks; I immediately acquainted the Medical Commissioners of the same, and at six in the evening, Mr. Hernandez, accompanied by Mr. Diez, surgeon, came and informed me, that his companion could not come by reason of his occupation, when the aforesaid Mr. Diez inoculated Nicholas Sanz, with matter taken from the goat, a child between two and three years of age, son of Nicholas and Isidora, living in Angel Street, in the yard belonging to the Mill House; and Maria, (whose mother's name is Michaela, and her husband, Mr. John Curial) a child eight months old, who live in St. Bernabe's Street. The inoculation was made in the arm, and both mothers declared their respective children never had the small-pox.

May 29. I went at nine in the morning to see the children that were inoculated, and found them without any symptoms of novelty.

May 30. I saw them at nine in the morning, but without any change.

May 31. At nine in the morning, I saw the child Nicholas, and was assured by his mother that he had a looseness in the night; this regularly proceeded from raw beans, which I found him eating the preceding days.

June 1. I discovered, at nine in the morning, two small pustules in both arms where the incisions for the inoculation had been made, which visibly proceeded from having been scratched, by which means the pocks that had been formed were destroyed. I observed nothing in the child Mary.

June 2. At nine in the morning, I discovered the child, Nicholas, to have a slight fever; his mother assured me he had it all night; I observed also some round spots of a red colour on his arm, the sides of the pimples of the incisions were something more inflamed, having a sufficient moisture; the child was seen to scratch them, though the mother was told to hinder it by putting on him a jacket, which she neglected doing. As no new appearance was visible in the child Mary, it made me suspect that she might have already had the natural small-pox, and I was not deceived in my idea; for the wife of the goat-herd assured me of it

 afterwards afterwards, and also assured the gentlemen, Mr. Peter Hernandez, and Mr. Nicholas Diez, that the child really had had the natural small-pox, which was basely dissembled by the mother, on account of the gratification, which was afterwards confirmed to me by the aunt of the goat-herd, as also by the mother herself, and finally it is discovered she is not the daughter of Michaela, but a child of the Foundling Hospital.

June 3. The mother of the child Nicholas, called me at seven in the morning, telling me the child had a high fever all that night; indeed, I found him to have a very slight fever, and the spots which I observed before, were increased in size, and inflamed, of an erysipelas colour. I informed the gentlemen commissioners, Mr. Hernandez and Mr. Diez, who went to see him.

June 4. At nine o'clock in the morning, I found in the place of the red spots, some pimples with clear matter; (and desired the mother to hinder his scratching) which I also acquainted the gentleman commissioners with. At six in the evening, we found him sleeping in the court-yard belonging to the house, almost naked, at a time when it was tolerably cold, and awaking him, found him without any fever; the pimples, that were scratched, were formed into pustules, and some food being given to him, he eat it with an appetite.

June 5. At nine in the morning, the pustules were something red without any fever.

June 6. Ibid.

June 7. The pustules and incrustated surfaces were almost dry.

June 8. The pustules and incrustated surfaces have disappeared. I continued to visit the two children twice every week till the 28th of June, without discovering any change, neither could any discovery be made of their infirmity or indisposition, either from the children themselves, or from any observations of their parents. That it produces less change than the vaccine pocks, proves incontestably the simplicity and benignity of the goat pocks. Nothing new has been observed with respect to the child, Maria, having had the small-pox before; the child Nicholas, notwithstanding the little attention paid to him by his mother, and the rigor of the season, as it was tolerably cold at that time, walked about naked, making use of eatables not conducive to health, and experienced nothing more than a slight fever from the fifth to the seventh day, in which the bladders and pustules appeared; they did not suppurate from the circumstance of their having been scratched off; and from the twelfth nothing new has been observed.

N. B. His Majesty the King of Spain has given orders that the children in the Foundling Hospital, and charity school dependant on the same, be inoculated with the goat pocks; of the former forty have lately been inoculated.

